# Essential role of polymerases for assay performance – Impact of polymerase replacement in a well-established assay

**DOI:** 10.1016/j.bdq.2018.10.002

**Published:** 2018-11-14

**Authors:** Anna Kristina Witte, Romana Sickha, Patrick Mester, Susanne Fister, Dagmar Schoder, Peter Rossmanith

**Affiliations:** a Christian Doppler Laboratory for Monitoring of Microbial Contaminants, Department of Veterinary Public Health and Food Science, University of Veterinary Medicine, Veterinaerplatz 1, 1210, Vienna, Austria; b Institute of Milk Hygiene, Milk Technology and Food Science, Department of Veterinary Public Health and Food Science, University of Veterinary Medicine, Veterinaerplatz 1, 1210, Vienna, Austria

**Keywords:** Polymerase chain reaction, qPCR, *Taq* polymerase, Poisson distribution, PCR-Stop analysis, qPCR validation

## Abstract

The quantitative real-time polymerase chain reaction (qPCR) is one of the most commonly molecular methods used today. It is central to numerous assays that have since been developed and described around its optimization. The *Listeria monocytogenes prfA* qPCR assay has been studied in great detail and due to its comprehensive knowledge, excellent performance (sensitivity of one single copy), and internal amplification control, it represents a suitable test platform for qPCR examinations. In this study, we compared ten different polymerases (or ready-to-use mastermixes) as possible (economic) alternatives to our gold standard Platinum *Taq* polymerase. We sought to determine the reproducibility of these assays under modified conditions, which are realistic because published assays are frequently used with substituted polymerases. Surprisingly, there was no amplification at all with some of the tested polymerases, even although the internal amplification control worked well. Since adaptation of the thermal profile and of MgCl_2_ concentration could restore amplification, simple replacement of the polymerase can destroy a well-established assay leading up to >10^6^-fold less analytical sensitivity. Further, validation using Poisson and PCR-Stop analyses revealed limits to some assay-polymerase combinations and emphasize the importance of validation.

## 1 Introduction

Ever since the first description of the polymerase chain reaction (PCR) at the beginning of the 1980s by Kary Mullis, many researchers have improved upon this method to the extent that it is now applicable to various fields of research and diagnostics. Development of the polymerase necessary for amplification in PCR moved from its origin in *Escherichia coli* towards heat stable polymerases that have the advantage of withstanding the high temperatures encountered with DNA denaturation [1]. *Taq* polymerase from *Thermophilus aquaticus* is now the predominantly used polymerase, but there are also other heat-stable variants available, such as *Pfu*, *Tfl* and *Tth* (*Pyrococcus furiosus*, *Thermus flavus* and *Thermus thermophilis*) [2]. Polymerases are often offered by various suppliers with hot start properties to avoid undesired enzymatic activity before the initial denaturation step. Chemical modifications or specific antibodies can be used to achieve this function [2].

For quantitative detection of DNA targets (quantitative PCR (qPCR)), fluorescent agents intercalating in double stranded DNA, such as SYBR or EVA green or fluorophores coupled to sequence-specific oligonucleotides can be detected by dedicated instruments [3]. In principle, due to its high sensitivity, qPCR is able to detect down to one initial target molecule number (ITMN) under optimized conditions as demonstrated for the probe-based *prfA* assay using Poisson distribution in the boundary limit (< 10 ITMN) [[4], [5], [6]].

*prfA* is a single copy locus of the foodborne pathogen *Listeria monocytogenes,* which causes listeriosis mainly in the immunocompromised with a high mortality rate [7,8]. As a consequence, it is strictly controlled by food safety and health agencies. In the EU, a microbiological zero tolerance criterion is in place for this organism for ready-to-eat foods [9]. In addition to classic microbiological detection methods, the *prfA* qPCR has been developed for detection of *L. monocytogenes* [10]. Besides its high sensitivity and reliability, the *prfA* assay is advantaged by its excellent internal amplification control (IAC) using the same primers but different probe from the original assay [11]. Moreover, the *prfA* assay has been tested and optimized for droplet digital PCR (ddPCR). For this application, the assay requires different amplification conditions, which have been comprehensively studied [6,12]. ddPCR is a relatively new PCR method based on Poisson distribution and permits quantification without external standards [13,14]. For all of these reasons, the *prfA* assay is ideal for evaluation of qPCR and related investigations.

We examined the performance of ten commercially available polymerases or mastermixes ranging from less costly to the expensive using the *prfA* assay. They were compared to our routinely used Platinum® *Taq* DNA Polymerase (Fisher Scientific), which is complexed with an antibody for its hot start property [15]. While the crucial role of the polymerase, as the central enzyme of every PCR, is universally acknowledged, its actual influence is frequently neglected when published assays are used. Therefore, we focused on the question of the transferability of such assays when the polymerase is replaced. Is the assay still reliable and does its analytical sensitivity remain identical? Are the published conditions usable? While there already exist a framework for presenting qPCR data to improve qPCR transparency and reliability, the *MIQE* (Minimum Information for Publication of Quantitative Real-Time PCR Experiments) guidelines [16], the aim of this study was to increase the trustfulness and reliability of qPCR data by demonstration of problems and solutions when (published) assay were used with other polymerases.

**Fig. 1:**
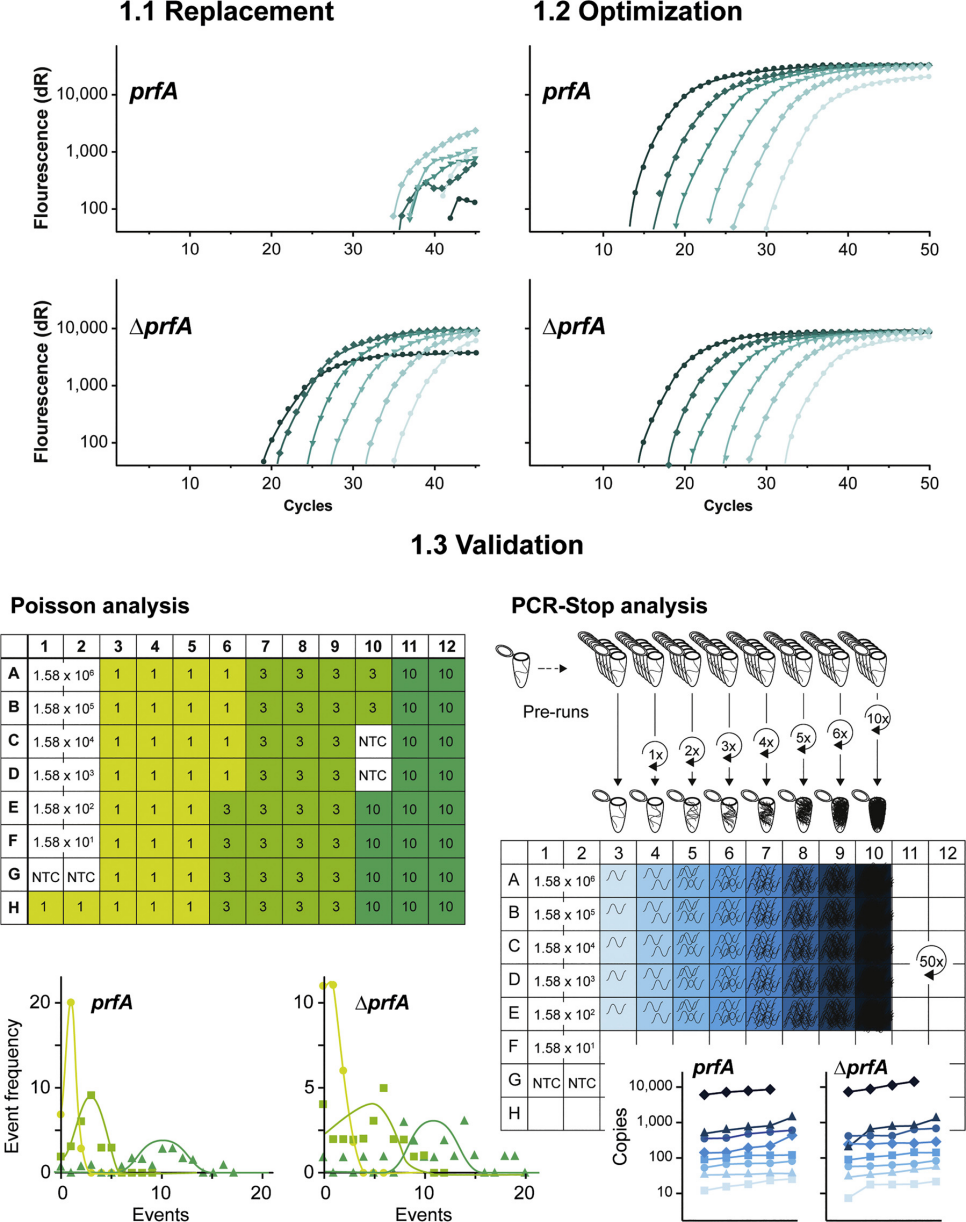
Test process of polymerases by the example of Probe/ROX qPCR Master Mix. 1.1 *prfA* and Δ*prfA* assays were initially carried out under the thermal profile of the established *prfA* assay (MgCl_2_ concentration as recommended by the supplier). 1.2 Afterwards, assays were adapted for amplification of all DNA concentrations of the calibration curve (1.58 × 10^1^-1.58 × 10^6^). 1.3 Finally, assays were validated using Poisson and PCR-Stop analysis (modified after [21]).

The data presented here showed that instead of simply replacing the enzyme, assays have to be optimized because of low performance under *prfA-standard-conditions*. It is remarkable that very different working conditions are necessary for each enzyme. Afterwards, assay parameters were investigated using Poisson and PCR-Stop analysis (Fig. 1). Interestingly, not all polymerases passed the validation methods in the *prfA* assay, but succeeded in the IAC assays, demonstrating the individual features for the assays despite the usage of the same primers.

## 2 Results

### 2.1 Comparison of calibration curves using different polymerases

For initial comparison of the different polymerases, DNA from *Listeria monocytogenes* EGDe and Δ*prfA* were amplified at concentrations between 1.58 × 10^1^ and 1.58 × 10^6^ copies per reaction, which are normally used for calibration curves for quantification of *L*. *monocytogenes*. Five ready-to-use-mastermixes (subsequently referred to as mastermixes, all except one intended for qPCR) and five standalone polymerases (subsequently referred to as polymerases), which have the 5′-3′ exonuclease activity necessary for probe degradation and are all except one less expensive alternatives as the mastermixes, were compared to our routinely used Platinum *Taq*. In a first attempt, qPCR was performed with the thermal program optimized for the assay using Platinum *Taq* (subsequently referred to as *prfA-standard-condition)*. As for the established *prfA* assay no additional reference dye was included and analysis is carried out with (not normalized) adaptive baseline correction by MxPro software, the same analysis was used for all polymerases/mastermixes independent whether or not they already included a reference dye. For the chemically-modified polymerase AmpliTaq Gold, a ten-minute denaturation step is necessary for activation [17] and therefore it was applied under the *prfA-standard-conditions* but with an extended initial denaturation step.

**Fig. 2:**
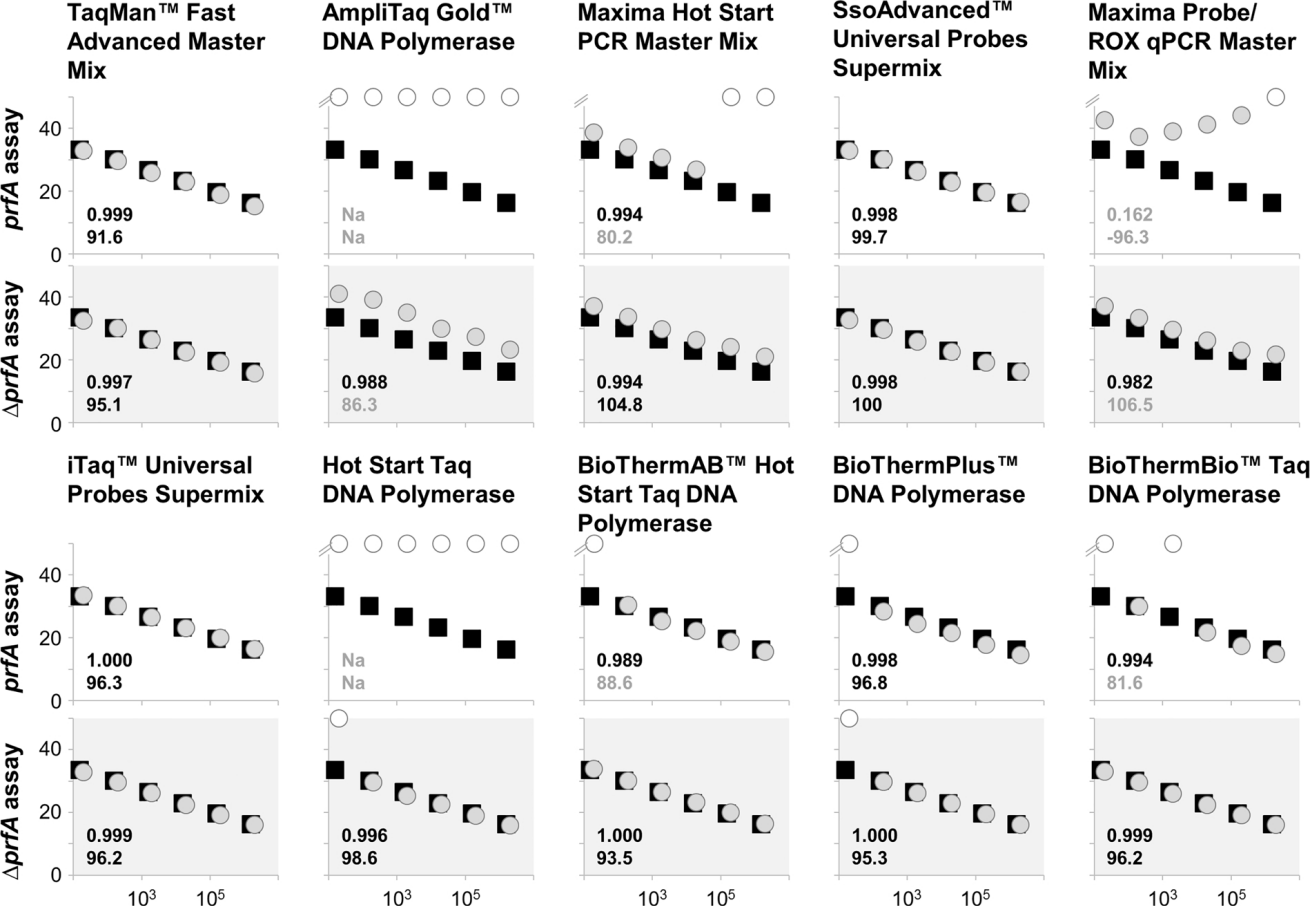
Amplification of *Listeria monocytogenes* EGDe and Δ*prfA* DNA using various polymerases under *prfA-standard-conditions*. Calibration curves (ranging from 1.58 × 10^1^ to 1.58 × 10^6^ ITMN per reaction, copies on the x-axis and Cq on y-axis) amplified under *prfA-standard-conditions* (only AmpliTaq Gold 10 min denaturation) with different polymerases/mastermixes (grey circles) were compared with the calibration curve amplified by Platinum *Taq* polymerase (black squares). All duplex reactions were displayed on top of each other with the white background for the *prfA* assay and grey for the Δ*prfA* assay. Rsq values and efficiencies (in %) were indicated for each polymerase/mastermix in the respective graph with Rsq values <0.98 and efficiencies more than 105% and less than 90% presented in grey. Rsq and efficiency for Platinum *Taq* polymerase are 1.000 and 96.7% in the *prfA* assay and 1.000 and 94% in the Δ*prfA* assay. No Cq were represented with white circles. One of two independent experiments including each six standards in single reactions is demonstrated. All PCRs except AmpliTaq Gold were carried out in the same run.

Two phenomena became immediately apparent (Fig. 2, Supplemental Table S1): Firstly, there are severe differences between the polymerases in terms of qPCR performance, and secondly, the effect is much more distinct in the *prfA* assay than in the Δ*prfA* assay. The latter effect is in agreement with data obtained in ddPCR, where the Δ*prfA* assay was less sensitive to assay modifications than the *prfA* assay [12]. In the *prfA* assay, only three of the ten polymerases/mastermixes amplified the complete DNA range of the calibration curve, while eight of the ten succeeded in the Δ*prfA* assay. In the *prfA* assay, the AmpliTaq Gold and Hot Start *Taq* failed completely (“no Cq”; Cq = quantification cycle: cycle that reaches the fluorescence threshold [16]) and results of the Maxima Probe/ROX qPCR Master Mix were problematic because respective Cq-values were all above 37 and incorrectly ranged (Fig. 1, 1.1).

Interestingly, the Rsq value (indicating the linearity of the PCR, theoretically 1 [18]) and efficiency (indicating the duplication of each cycle, theoretically 100% [16]) were frequently, but not always, affected in samples where the lowest DNA concentration was not amplified. For example, in the case of the Δ*prfA* assay, the Rsq was 1 and the efficiency 95.3% (Platinum *Taq*: 1; 94%) on amplification with BioThermPlus, while the lowest DNA concentration (1.58 × 10^1^) could not be detected. This shows that efficiency and Rsq values solely are not the best criteria for assay valuation and it is important to include analytical sensitivity. This parameter is regularly expressed as limit of detection (LOD) [16]. The results of this study demonstrate that under established conditions different polymerases lead to 10-fold or even a 10^6^-fold decrease in sensitivity.

Further, the amplification curves of most of the polymerases did not appear optimal and the maximum signal (difference between background fluorescence and maximum fluorescence) varied considerably, especially in the *prfA* assay (Fig. 2, Supplemental Figure S2). This indicates that modifications are necessary for optimal results as demonstrated similarly for ddPCR [12]. It also shows that a well-established assay must be re-adapted and validated when polymerases are replaced.

### 2.2 Adaptations

**Fig. 3:**
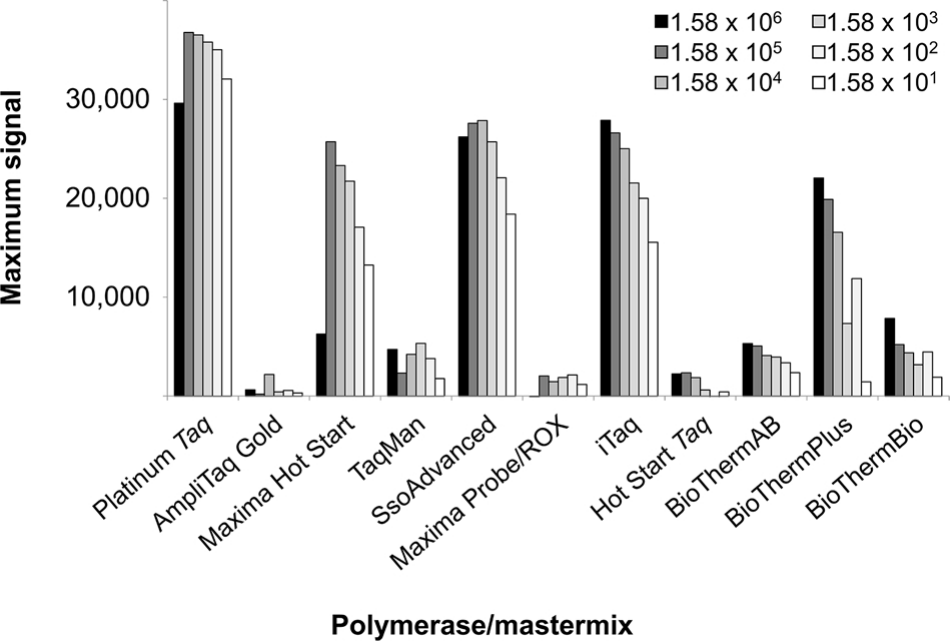
Maximum signals under *prfA-standard-conditions* for the *prfA* assay. The maximum signals (difference between maximum fluorescence and background fluorescence) vary under *prfA-standard-conditions* depending upon the polymerase used. One of two independent experiments including each six standards in single reactions is demonstrated. All PCRs except AmpliTaq Gold were carried out in the same run.

**Fig. 4:**
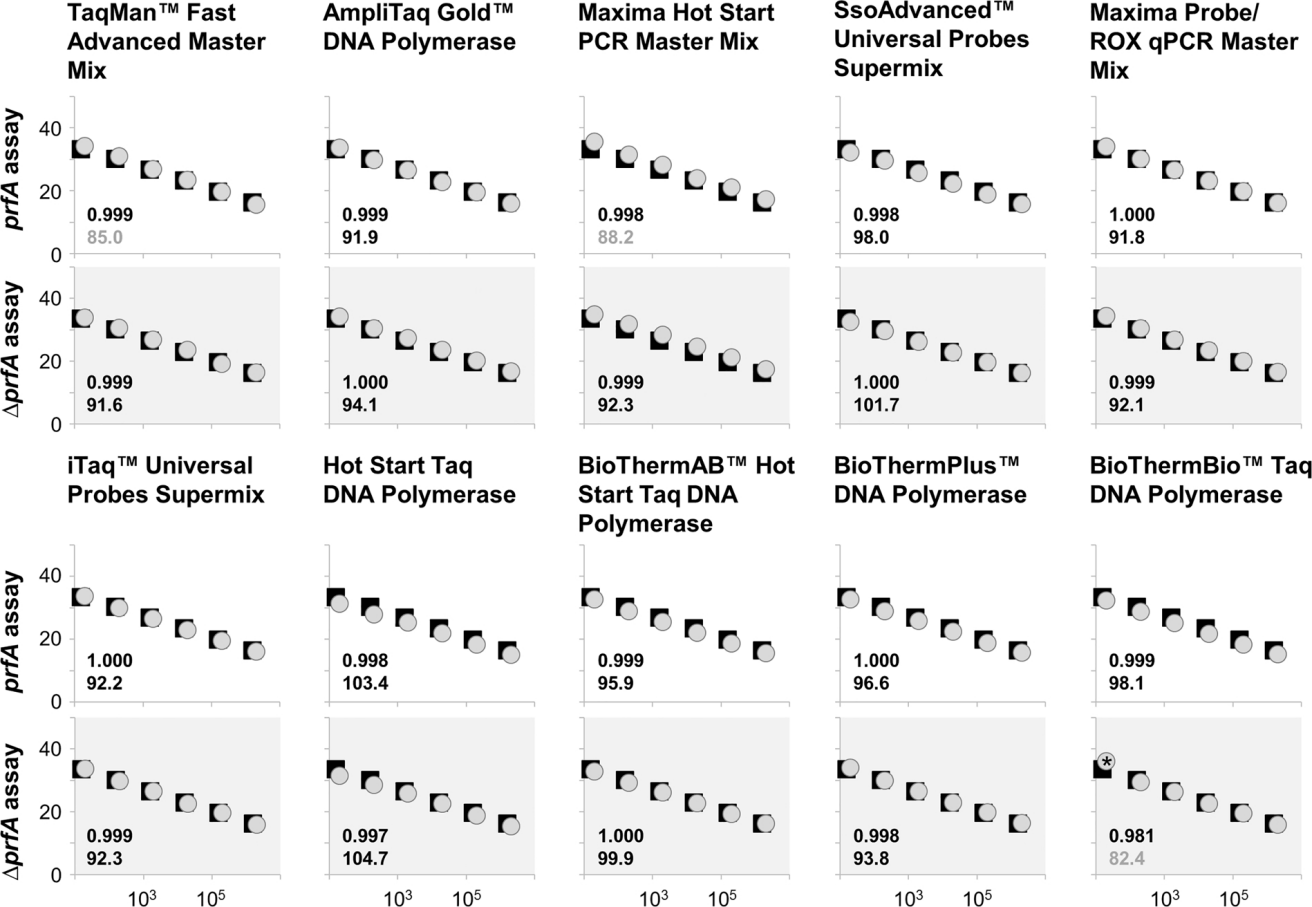
Amplification of *Listeria monocytogenes* EGDe and Δ*prfA* DNA using various polymerases under optimized conditions. Calibration curves (ranging from 1.58 × 10^1^ to 1.58 × 10^6^ ITMN per reaction, copies on the x-axis and Cq on y-axis) amplified under optimized conditions with different polymerases/mastermixes (grey circles) were compared with the calibration curve amplified by Platinum *Taq* polymerase (black squares). All duplex reactions were displayed on top of each other with the white background for the *prfA* assay and grey for the Δ*prfA* assay. Rsq values and efficiencies (in %) were indicated for each polymerase/mastermix in the respective graph with Rsq values <0.98 and efficiencies more than 105% and less than 90% presented in grey. Rsq and efficiency for Platinum *Taq* polymerase are 1.000 and 96.7% in the *prfA* assay and 1.000 and 94% in the Δ*prfA* assay. One of two independent experiments including each six standards in single reactions is demonstrated (* was in repetition “no Cq”)). All PCRs with the same thermal profile were carried out in the same run.

For assay optimizations, physical and chemical parameters such as the thermal profile (times and temperatures), the concentrations of chemicals (primer, probes, MgCl_2_, etc.) and, of course, combinations of both can be adapted. As with Fachmann and colleagues [19], we originally desired solely a cost-effective polymerase exchange without performing too many optimizations other than specific necessities such as the initial denaturation of the AmpliTaq Gold. However, this extended denaturation step appears to be insufficient for satisfactory amplification and the performance of most other polymerases was also poor (Fig. 2, Supplemental Table S1). These observations prompted us to perform some further adaptations/optimizations. Our goal was to obtain the optimum conditions for all polymerases. Indeed, we identified conditions for all polymerases, where amplification of all DNA concentrations of the calibration curve was successful and Cq values were in the same range as the ones of Platinum *Taq* polymerase. Furthermore, efficiencies and Rsq values should be at least as acceptable as in literature described (efficiency 90–105%, Rsq>0.98 [20]) and maximum signals more equalized (Fig. 4, Fig. 3, Supplemental Table S3, Supplemental Figure S2). Only the BioThermBio signal was instable when using the lowest DNA standard (15 initial target molecule numbers (ITMN)), even although the Cq-values of the complete calibration curve matched with those of the remaining polymerases (Fig. 4, Supplemental Table S3).

In a previous study investigating *prfA* ddPCR optimization, extension of the denaturation and elongation steps and using a temperature of 59 °C for combined annealing/elongation was found to be optimum for the polymerase in the ddPCR mastermix [12]. Indeed, the iTaq and SsoAdvanced seem to work slightly better under those conditions, although both these polymerases are already among the most satisfactory under *prfA-standard-conditions*, which are advantaged by cycling faster. Adaptation to the “ddPCR” thermal profile was the most optimum profile for AmpliTaq Gold. In summary, different conditions were required depending upon the polymerase. Hot Start *Taq*, BioThermBio, BioThermAB and BioThermPlus required supplementary MgCl_2_ in addition to the buffer that already contains the recommended amount of MgCl_2_ for these polymerases. The other polymerases and the mastermixes could be adjusted only by adapting the thermal profile. Some polymerases/mastermixes (AmpliTaq Gold, SsoAdvanced and iTaq) performed well with a combined denaturation/elongation step while others worked better using separate denaturation and elongation steps (Taqman, Maxima Hot Start, Maxima Probe/Rox, Hot Start *Taq*, BioThermBio, BioThermAB and BioThermPlus). In contrast to a study with ddPCR [12], and some of the tested polymerases of that study, all BioTherm polymerases performed better with fast cycling protocols. Optimized working conditions for all polymerases are summarized in Table 1.

**Table 1:** Optimized conditions for *prfA* qPCR.

Polymerase	Initial denaturation [°C – min]	Denaturation [°C – min]	Annealing/ Annealing-Elongation [°C – min]	Elongation [°C – min]	Cycles	MgCl_2_ [mM]
*prfA-standard-conditions*	94 - 2.00	94 - 0.15	64 - 1.00	–	45	
Platinum™*Taq* DNA Polymerase	94 - 2.00	94 - 0.15	64 - 1.00	–	45	3.5
TaqMan™ Fast Advanced Master Mix	95 - 5.00	95 - 0.30	57 - 0.30	72 - 1.00	50	(buffer)
AmpliTaq Gold™ DNA Polymerase	95 - 10.00	94 - 1.00	59 - 2.00	–	50	1.5 (buffer)
Maxima Hot Start PCR Master Mix	95 - 5.00	95 - 0.30	57 - 0.30	72 - 1.00	50	2 (buffer)
SsoAdvanced™ Universal Probes Supermix	94 - 2.00 (95 - 10.00)	94 - 0.15 (94 - 1.00)	64 - 1.00 (59 - 2.00)	– (-)	45 (50)	(buffer)
Maxima Probe/ROX qPCR Master Mix	95 - 7.00	95 - 0.30	55 - 0.30	72 - 1.30	50	(buffer)
iTaq™ Universal Probes Supermix	94 - 2.00 (95 - 10.00)	94 - 0.15 (94 - 1.00)	64 - 1.00 (59 - 2.00)	– (-)	45 (50)	(buffer)
Hot Start *Taq* DNA Polymerase	95 - 2.00	95 - 0.15	51 - 0.30	68 - 1.00	50	4.5
BioThermAB™ Hot Start Taq DNA Polymerase	95 - 3.00	95 - 0.30	57 - 0.30	72 - 1.00	50	3.5
BioThermPlus™ DNA Polymerase	95 - 2.00	95 - 0.20	51 - 0.20	72 - 0.30	50	2.5
BioThermBio™ Taq DNA Polymerase	95 - 2.00	95 - 0.20	51 - 0.20	72 - 0.30	50	2.5

Maximum signals are summarized in Supplemental Figure S4 and demonstrate that there are minor differences on using higher DNA concentrations under optimized conditions, but still distinct variances by amplification of a smaller target number. That means either that the conditions are still not optimal for the polymerases or that a particular limit for the polymerase is reached.

In accordance with previous results, it became obvious during adaptations that in comparison to the *prfA* assay, the Δ*prfA* assay was more robust and performed satisfactorily under more conditions. Both PCR products were amplified with identical primers having similar melting temperatures (*prfA* 77.9 °C and Δ*prfA* 78.3 °C). Nevertheless, the GC content is slightly different whereby the product of the Δ*prfA* assay is with 49% close to the general rule of having optimally 50% while the one of the *prfA* assay is lower with 37%. The length of the amplicon highlights the main difference (274 bp and 100 bp) [12]. However, as all polymerases used are suitable for amplification of longer PCR products, it remains unclear whether length is really the cause for the observed distinct differences.

### 2.3 Validation using Poisson distribution and PCR-Stop analysis

For assay validation, the assay performance parameters, quantitative and qualitative limits as well as resolution for each optimized assay were tested to determine for which applications the polymerase could be used. The boundary limit area lower than 10 ITMN was proved using the Poisson distribution as described previously [[4], [5], [6]]. Further, to check qPCR performance during initial cycles and the quantitative resolution, PCR-stop analysis was performed using 10 ITMN as template [21]. These tests for assay validation were carried out with the most optimal conditions for each polymerase.

More than half of the tested polymerases produced satisfactory results with Poisson analysis for both *prfA* and Δ*prfA* assays suggesting a detection limit of one single ITMN. BioThermBio, BioThermPlus and Hot Start *Taq* produced satisfactory results with the Δ*prfA* assay but not with the *prfA* assay. BioThermBio could not amplify 10 ITMN at all (Supplemental Table S5).

In the PCR-Stop analysis, the performance of most polymerases resulted in good resolution and deviations within the batches were minor (Supplemental Table S6). However, a few of the tested enzymes performed poorer than the others: BioThermBio did not show any amplification with 10 ITMN in the *prfA* assay, which is not surprising since the amplification of the lowest standard DNA concentration was already sporadic (Fig. 4, Supplemental Table 3). However, the simultaneous Δ*prfA* assay was acceptable. Similar results were obtained with BioThermPlus. The Taqman mastermix showed poor performance in the *prfA* assay but better (but not excellent) in the Δ*prfA* assay. These results are rather surprising since the Poisson analysis for both assays was adequate. However, the cumulative occurrence of negative samples in the “10 ITMN batch” in the Poisson analysis, which was the main reason preventing excellence, has already indicated that the assay is suboptimal. Hot Start *Taq* showed a rather high occurrence of negative samples, especially in the *prfA* assay, indicating a qualitative limit >10 ITMN, which is in agreement with the Poisson analysis.

In summary, the Δ*prfA* assay passed both validation methods with all polymerases. In contrast, BioThermBio, BioThermPlus, Hot Start *Taq* failed Poisson and PCR-Stop analyses in the *prfA* assay and Taqman mastermix only PCR-Stop analysis.

## 3 Discussion

In this study, ten commercially available polymerases and mastermixes were compared by means of the *prfA* and Δ*prfA* assays with Platinum *Taq,* which is commonly used for these assays. Results demonstrate that straightforward transferability of the assay qualities is not given since severe differences between all polymerases and the two assays were detected. However, quite extensive adaptations could restore assay qualities for most polymerases. Yet, differences were not confined to polymerase-specific characteristics, such as chemical or antibody inactivated hot starts. Furthermore, this study demonstrated that adaptation is still necessary when qPCR mastermixes are used. As the mastermixes, in contrast to standalone polymerases do normally not need MgCl_2_ adjustments, adaptations are less cumbersome. This could probably also explain, that the first trial using *prfA-standard-conditions* was probably more successful for qPCR mastermixes than for the standalone polymerases. In this context, it is important to mention that missing fluorescent signal in qPCR could also be caused by low MgCl_2_ concentrations resulting in inefficient probe binding or cleavage [22]. However, in the cases presented in this study, this scenario is less possible since the control reaction (Δ*prfA* assay, also with probe) was successful under most conditions and Cq values in the *prfA* assay were not increased but analytical sensitivity was diminished. Furthermore, *prfA* products were not detected in all samples on agarose gels.

Other studies have shown that variant polymerases react differently to PCR inhibitors [[23], [24], [25]] and therefore engineering of polymerases with enhanced resistance to environmental inhibitors has been investigated [26]. However, the effect reported in the present study is considered unlikely to be inhibitor-related because, first of all, DNA was extracted from pure cultures with an extraction kit including two washing steps. Further, the low DNA quantities caused more variations than high DNA concentrations. Since the low DNA concentrations were at least 1:10^6^ dilutions of the original extracted DNA, the possibility of inhibitors can be excluded. In addition, adaptation of the thermal profile could at least partially restore polymerase performance. Undoubtedly, the tested polymerases might have differed in respect of their sensitivity to inhibitors, but this was not focus of this study. Nevertheless, this point should be investigated further when this assay is used for detection of *Listeria monocytogenes* in foodstuffs, since food matrices often contain inhibitors [27].

The performance differences of various polymerases and mastermixes have been examined before. The disparity of five polymerases regarding efficiency and the detection window has been examined with *Yersinia* assays [28]. Buzard and colleagues compared ten different qPCR mastermixes with four bacterial assays and demonstrated differences between the mastermixes and assays [29]. Additionally, Fachmann et al., for cost-effective qPCR optimization, compared 16 polymerases with two bacterial assays and different sample types and concluded that the choice of the appropriate polymerase depends upon the assay, question and sample type [19]. Likewise, unequal performances of diverse assays using different mastermixes were demonstrated recently [30]. However, none of those studies tested whether adaptations can restore assay performance.

The current study demonstrated that such comparisons are indeed realistic but not entirely fair, because the tested conditions were adapted to another specific polymerase. Results reflect the differences found in the other studies while also offering a solution. Moreover, the present study compares exclusively Taq polymerases (only BioThermPlus is a mixture of *Taq* and *Pfu* polymerase and for SsoAdvanced no information is provided), while the other studies also included other polymerase types which could additionally influence results. In addition, the performance parameters were thoroughly investigated to figure out the limits of each polymerase in the *prfA* and Δ*prfA* assays. Together with the studies mentioned, our findings indicate that some assays are more “resistant” to polymerase changes than others.

Successful qPCR conditions were almost always different between polymerases and mastermixes tested in this study. Unfortunately, we cannot provide a universal strategy to modify published assays when replacing the enzyme. As rough orientation for standalone polymerases, we started with the original protocol using the suppliers recommended MgCl_2_ concentration and (at best simultaneously) with the one of the original protocol. Next, the optimal polymerase amount was verified and further MgCl_2_ concentrations were tested. The thermal profile for polymerases and mastermixes was modified according to suggestions from either by the suppliers, from other publications and from our own in-house experience. As the procedure might be tedious and costly, a possibility to reduce the amount of PCR reactions (and thus expenditure), is the exclusive usage of the lower standard concentrations, which are more critical, instead of using the complete calibration curve.

Although well-established, reliable and sensitive when used under “original” conditions, the *prfA* assay is more sensitive to enzyme exchange. This might be a reason why this assay is rarely performed by other research groups. The *prfA* assay is published with the use of Platinum *Taq*. This enzyme proved to be very stable in this study and worked satisfactorily in most tested conditions, but it is also the most expensive. Since costs are an important factor in research and application, this enzyme is probably not often used for routine work. Consequently, when this published *prfA* assay is implemented under published conditions, the qPCR might completely fail or appear insufficient when another polymerase than the Platinum *Taq* is used. In practice, results demonstrate that the polymerase used in a well-established system is not simply interchangeable. In contrast, the Δ*prfA* assay is much more stable and resilient. As discussed in the context of ddPCR [12], the main difference compared with the *prfA* assay is the shorter length and the sequence as such, since the primers are identical. Furthermore, the higher GC content of the Δ*prfA* assay’s PCR product enhances stability. Of course, different sequences effect different hybridization properties and, as general rule, shorter PCR products are more efficient than longer [30]. However, under the conditions used, both assays have been equally efficient for many years. Nevertheless, it remains unclear which of the factors are responsible for the different behavior. The different responses to changes of both assays emphasize the specific behavior of each assay despite high similarities.

The polymerases with the poorest performance are mainly the less expensive ones. However, the Taqman mastermix, which was unsatisfactory in PCR-Stop (*prfA*), is one of the most expensive. In contrast, BioThermAB is rather inexpensive but showed good performances. The middle-price segment, including iTaq, Maxima Probe/ROX qPCR Master Mix and SsoAdvanced, also performed well and might be suitable alternatives. Thus, the performance does not always correlate with the price. To summarize, we generated a grade for performance and price for the polymerases used in this study (Table 2). Nevertheless, the value of this ranking will most likely vary with differing assays and whether performance or economy has foremost importance is depending on the question.

**Table 2:** Price and performance rating.

			Performances			
	Price	Average performance	*prfA* Poisson	Δ*prfA* Poisson	*prfA* PCR-Stop	Δ*prfA* PCR-Stop
**Platinum™*Taq* DNA Polymerase**	1	5	5	5	5	5
**TaqMan™ Fast Advanced Master Mix**	2	3	3	4	2	3
**AmpliTaq Gold™ DNA Polymerase**	2	4.5	4	4	5	5
**Maxima Hot Start PCR Master Mix**	2	4.75	5	5	4	5
**SsoAdvanced™ Universal Probes Supermix**	3	4.25	4	4	4	5
**Maxima Probe/ROX qPCR Master Mix**	3	4.25	4	4	5	4
**iTaq™ Universal Probes Supermix**	3	4.25	4	4	5	4
**Hot Start *Taq* DNA Polymerase**	3	3	2	3	3	4
**BioThermAB™ Hot Start Taq DNA Polymerase**	4	4.25	4	4	3	5
**BioThermPlus™ DNA Polymerase**	4	2.5	2	3	1	3
**BioThermBio™ Taq DNA Polymerase**	5	2.25	1	4	1	3

Price categories (price [€] per reaction): 5: very inexpensive (< 0.20); 4: inexpensive (0.21 – 0.40); 3: medium-priced (0.41 - 0.60); 2: expensive (0.61 - 0.99); 1: very expensive (> 1.00).

Performance categories: 5: excellent; 4: good; 3: average; 2: fair; 1: poor.

## 4 Conclusions

The spectrum of commercially polymerases is huge and accordingly each polymerase is different in respect of performance and conditions required. This study shows that simple replacement of the polymerase in a well validated assay can actually destroy it without necessary adaptations. Otherwise, there is a risk of reduced assay performance and, after adaptations are implemented, validation and specificity should be verified. On the other hand, data also suggest that some assays might perform better than anticipated when they are modified or other polymerases are used. In common with other investigators, we use the polymerase available in our laboratory for new assays and simply order new primers and probes. However, the presented data demonstrate that the transferability of an assay simply by the exchange of the polymerase is not without problems. This should be heeded when applying assays from other publications. This is especially true for low DNA target concentrations and precise quantification purposes, which necessitate proper validation. Moreover, results highlight that calibration curves alone are not sufficient for validation and methods such as Poisson and PCR-Stop analysis are essential.

In summary, the assay quality, reliability and sensitivity is not given by replacement of polymerase and we wish to encourage other scientist to focus more on this enzyme when developing new assays or when using published assays to improve qPCR trustfulness and reliability. Moreover, it should be considered that many publications might already be affected of such phenomenon.

## 5 Methods

### 5.1 DNA isolation

DNA was isolated using the NucleoSpin tissue kit (Macherey Nagel, Düren, Germany) following protocol instructions for Gram-positive bacteria. The DNA was eluted twice with 50 μl ddH_2_O (70 °C).

### 5.2 DNA standard for real-time PCR quantification

One milliliter of a L. *monocytogenes* (strain EGDe 1/2a) or Δ*prfA* L. *monocytogenes* (strain EGDe 1/2a [11], both part of the collection of bacterial strains at the Institute of Milk Hygiene, Milk Technology and Food Science, University of Veterinary Medicine, Vienna, Austria) overnight culture (grown in tryptone soya broth with 0.6% (w/v) yeast extract (TSB-Y; Oxoid, Hampshire, UK) at 37 °C) was used for DNA isolation. The DNA concentration was measured with the Qubit dsDNA Broad Range Kit (Fisher Scientific, Vienna, Austria). The copy number of the single-copy *prfA* gene was calculated using the molecular weight (1 ng of DNA equals 3.1 × 10^5^ copies of the genome).

### 5.3 qPCR. “established Standard qPCR”

One qPCR of 25 μl final volume contained 2.5 μl 10 × reaction buffer (Fisher Scientific, Vienna, Austria), 3.5 mM MgCl_2_,12.5 pmol of each primer (Table 3), 6.25 pmol of each probe (Table 3), 5 nmol each dATP, dTTP, dGPT, and dCTP, 1.5 U of Platinum Taq (Fisher Scientific, Vienna, Austria), and 2.5 μl of each template DNA. The *prfA* qPCR was performed as previously published in an Mx3000p real-time PCR thermocycler (Stratagene, CA, USA) with initial denaturation at 94 °C for 2 min, amplification in 45 cycles at 94 °C for 15 s and 64 °C for 1 min [31] (*prfA-standard-conditions*). The data were analyzed with MxPro software (adaptive baseline settings).

**Table 3:** Primers and probes.

name	sequence	
LIP1	5`-GAT ACA GAA ACA TCG GTT GGC-3`	(Eurofins, Ebersberg, Germany)
LIP2	5`-GTG TAA TCT TGA TGC CAT CAG G-3`	(Eurofins, Ebersberg, Germany)
LIP probe2	5`-FAM-CAG GAT TAA AAG TTG ACC GCA-MGB-3`	(Fisher Scientific, Vienna, Austria)
LIP probe2*	5`-FAM-CAG GAT TAA AAG TTG ACC GCA-BHQ1-3`	(Eurofins, Ebersberg, Germany)
p-lucLm 5	5`-HEX-TTC GAA ATG TCC GTT CGG TTG GC-BHQ1-3`	(Eurofins, Ebersberg, Germany)

* due to financial reasons adaptations etc. were not performed with MGB-labeled probe. Results match those obtained with MGB-labeled probe (verified). Fluorescent signals, however, diverge and results were only compared to those with the same probe.

### 5.4 Other polymerases and mastermixes

The qPCR of the standalone polymerases was of 25 μl final volume containing their specific 10 x reaction buffer (2.5 μl), 12.5 pmol of each primer, 6.25 pmol of each probe, 5 nmol each dATP, dTTP, dGPT, and dCTP, 1.5 U of the polymerases (Table 4), respectively. The first qPCRs were performed without additional MgCl_2_ as suggested by the suppliers (buffers contain 1.5 mM MgCl_2_).

**Table 4:** Polymerases and mastermixes.

Name	Company	Price/ reaction*
Platinum™ *Taq* DNA Polymerase	Fisher Scientific, Vienna, Austria	1.34 €
TaqMan™ Fast Advanced Master Mix ^mq^	Fisher Scientific, Vienna, Austria	0.87 €
AmpliTaq Gold™ DNA Polymerase	Fisher Scientific, Vienna, Austria	0.73 €
Maxima Hot Start PCR Master Mix^m^	Fisher Scientific, Vienna, Austria	0.72 €
SsoAdvanced™ Universal Probes Supermix ^mq^	Bio-Rad, Munich, Germany	0.56 €
Maxima Probe/ROX qPCR Master Mix ^mq^	Fisher Scientific, Vienna, Austria	0.55 €
iTaq™ Universal Probes Supermix ^m,q^	Bio-Rad, Munich, Germany	0.49 €
Hot Start *Taq* DNA Polymerase	New England BioLabs, Frankfurt, Germany	0.40 €
BioThermAB™ Hot Start Taq DNA Polymerase	BioAxis Genecraft, Cologne, Germany	0.24 €
BioThermPlus™ DNA Polymerase	BioAxis Genecraft, Cologne, Germany	0.23 €
BioThermBio™ Taq DNA Polymerase	BioAxis Genecraft, Cologne, Germany	0.15 €

^m^ready-to- use-mastermix; ^q^for qPCR.

* calculated from 2017 price lists for the largest package size without discounts or promotions. For standalone polymerases 1.5 U per reaction were used and prices calculated accordingly.

For the ready-to-use mastermixes (in Table 4 labeled with ^m^) one qPCR reaction of 20 μl (TaqMan™ Fast Advanced Master Mix, SsoAdvanced™ Universal Probes Supermix and iTaq™ Universal Probes Supermix), 25 μl (Maxima Probe/ROX qPCR Master Mix) or 50 μl (Maxima Hot Start PCR Master Mix) final volume contained 10 μl (TaqMan, SsoAdvanced and iTaq), 12.5 μl (Maxima Probe/ROX) or 25 μl (Maxima Hot Start) mastermix, 12.5 pmol of each primer, 6.25 pmol of each probe, and 2.5 μl of each template DNA. qPCR was performed as described above. Deviations in MgCl_2_ concentrations and thermal profiles are described in the results section. The data were analyzed with MxPro software (adaptive baseline settings) without any reference dye normalization. Respective no template controls for each mastermix were included and were consistently negative (No Cq).

### 5.5 qPCR adaptations

Firstly, qPCR was performed with polymerases or mastermixes with the supplier’s recommended MgCl_2_ concentration using the *prfA-standard-program* and chemistry. In parallel or afterwards, polymerases were tested with the MgCl_2_ concentration optimal for Platinum *Taq* in the *prfA* assay (3.5 mM) as well as one MgCl_2_ concentration in between and verified that the usage of 1.5 U polymerase is indeed more efficient than lower concentrations. Afterwards, the thermal program was modified (for mastermixes and polymerases). For this purpose, the “ddPCR program” with extended times (and lower elongation/annealing temperature) and the suppliers’ recommended programs were used. If results were still not satisfying, further (lower) temperatures and combinations with different MgCl_2_ concentrations were tested. All adaptation conditions were carried out at least with three different DNA concentrations in single reactions (lowest standard concentrations: 1.58 × 10^1^, 1.58 × 10^2^ and 1.58 × 10^3^ copies per reaction). *prfA-standard-conditions* were repeated as independent repetition in the same run for all polymerases/mastermixes except AmpliTaq (to gain at least two independent runs) and optimized conditions were confirmed with an independent repetition on one day and furthermore confirmed later in the Poisson and PCR-stop analysis (to archive at least four independent runs).

### 5.6 Poisson distribution-based approach

The Poisson distribution-based approach was carried out as described previously [4,6] (Fig. 1): To receive one, three and ten initial target molecule numbers (ITMN), the DNA of the lowest log-scale standard (15 ITMN) was diluted, respectively. 30 qPCRs for one and three ITMN and 20 qPCRs for ten ITMN were performed and data were rounded mathematically. Means, distribution of values and ratio of positive/negative PCRs were analyzed according to Poisson distribution. When fitting data to a Poisson distribution, no other statistics were applied [4]. Unless inconsistencies of the controls (six standards in duplicates, four NTCs) appeared, Poisson analysis was performed once, whereby the experiment completed all 96 wells in the cycler including a standard curve comprising six standards in duplicate. For each polymerase, the most optimum thermal profile was used (Table 1).

### 5.7 PCR-Stop experiments

To monitor the performance of qPCR during the first cycles, PCR-Stop analysis was performed as described previously [21] (Fig. 1) with slight modification using 10 ITMN with five replicates. qPCRs with 1–6, 10 and without any pre-run were performed under the same thermal profile as the Poisson experiments. After the pre-runs, the tubes containing the PCR reaction were stored at 4 °C and started simultaneously. For analysis, results were illustrated in graphs (repetitions versus quantity) to examine the regularity within the batches and assay quantitative resolution [21]. Unless inconsistencies of the controls (six standards in duplicates, two NTCs) appeared, PCR-stop analysis was performed once.

## Competing interests

The authors declare that there are no conflicts interests.
